# *CaFtsH06*, A Novel Filamentous Thermosensitive Protease Gene, Is Involved in Heat, Salt, and Drought Stress Tolerance of Pepper (*Capsicum annuum* L.)

**DOI:** 10.3390/ijms22136953

**Published:** 2021-06-28

**Authors:** Jing-Jing Xiao, Rui-Xing Zhang, Abid Khan, Saeed ul Haq, Wen-Xian Gai, Zhen-Hui Gong

**Affiliations:** 1 College of Horticulture, Northwest A&F University, Yangling, Xianyang 712100, China; jingjingxiao136@163.com (J.-J.X.); xingqing@nwafu.edu.cn (R.-X.Z.); saeed_ulhaq@nwafu.edu.cn (S.u.H.); gaiwenxian@163.com (W.-X.G.); 2 Department of Horticulture, The University of Haripur, Haripur 22620, Pakistan; abidagriculturist@gmail.com; 3 Department of Horticulture, University of Agriculture Peshawar, Peshawar 25120, Pakistan

**Keywords:** *CaFtsH06*, *Capsicum annuum* L., transgenic *Arabidopsis*, abiotic stress, ROS-scavenging system

## Abstract

Harsh environmental factors have continuous negative effects on plant growth and development, leading to metabolic disruption and reduced plant productivity and quality. However, filamentation temperature-sensitive H protease (FtsH) plays a prominent role in helping plants to cope with these negative impacts. In the current study, we examined the transcriptional regulation of the *CaFtsH06* gene in the R9 thermo-tolerant pepper (*Capsicum annuum* L.) line. The results of qRT-PCR revealed that *CaFtsH06* expression was rapidly induced by abiotic stress treatments, including heat, salt, and drought. The CaFtsH06 protein was localized to the mitochondria and cell membrane. Additionally, silencing *CaFtsH06* increased the accumulation of malonaldehyde content, conductivity, hydrogen peroxide (H_2_O_2_) content, and the activity levels of superoxide dismutase and superoxide (·O_2_^−^), while total chlorophyll content decreased under these abiotic stresses. Furthermore, *CaFtsH06* ectopic expression enhanced tolerance to heat, salt, and drought stresses, thus decreasing malondialdehyde, proline, H_2_O_2_, and ·O_2_^−^ contents while superoxide dismutase activity and total chlorophyll content were increased in transgenic Arabidopsis. Similarly, the expression levels of other defense-related genes were much higher in the transgenic ectopic expression lines than WT plants. These results suggest that *CaFtsH06* confers abiotic stress tolerance in peppers by interfering with the physiological indices through reducing the accumulation of reactive oxygen species, inducing the activities of stress-related enzymes and regulating the transcription of defense-related genes, among other mechanisms. The results of this study suggest that *CaFtsH06* plays a very crucial role in the defense mechanisms of pepper plants to unfavorable environmental conditions and its regulatory network with other *CaFtsH* genes should be examined across variable environments.

## 1. Introduction

As sessile organisms, plants endure limitations to their growth, development, and agricultural productivity caused by abiotic stresses such as drought, salinity, and high temperatures [1,2,3]. Unfavorable circumstances produce a variety of morphological and physiological changes in plants, as well as considerable damage to membrane integrity and stability, which results in intracellular material exosmosis [1,4,5]. Plants have a number of defense mechanisms enabling their long-term acclimation to adverse environmental conditions, such as changes in the levels of phytohormones and Ca^2+^ content as well as reactive oxygen species (ROS) signaling and plant-programmed cell death (PCD) [6,7]. Under stress conditions, ROS are induced, which leads to oxidative stress, and, interestingly, plants have evolved sophisticated adaptive systems at the molecular level, including antioxidants enzymes [8].

Protein denaturation will occur as a result of the sustained high temperature, which may result in the loss of protein function. Under heat stress conditions, through the restoration and hydrolysis of protein regulation systems, these defense mechanisms work together to repair functional failure proteins to maintain normal metabolic mechanisms [9]. Filamentation temperature-sensitive H (FtsH), which has an N-terminal transmembrane domain followed by an AAA domain [10,11], is a member of the ATP-dependent protease hydrolysis system family, which has ATPase and molecular chaperone activity [12,13]. Additionally, FtsH protease activity can degrade misfolded and mistranslated proteins and provide a control mechanism to remove dysfunctional proteins in cells as central elements of energy metabolic control systems [14,15].

In higher plants, there are many members of the FtsH family. There are 12 *FtsH* genes in *Arabidopsis thaliana*, with three proteins (FtsH3, FtsH4, and FtsH10) localized to the mitochondria, eight (FtsH1, FtsH2, FtsH5 to FtsH9 and FtsH12) targeted to the chloroplasts, and one (FtsH11) entering both the mitochondria and chloroplasts [10,16,17]; these genes participate in a variety of abiotic stress responses such as those to heat shock, hypertonicity, light stress, and cold [18,19,20,21,22]. AtFtsH6 protease degrades Lhcb3 and Lhcb1 so as to participate in senescence and high light acclimation [23]. AtFtsH1 participates in the degradation of the 23 kDa peptide fragment of the D1 protein photooxidative damage product [24]. In spinach, FtsH appears to be involved in D1 protein turnover in response to heat stress [25].

FtsH is an energy-dependent protease that is required for the growth of many prokaryotes. The FtsH protease from *Escherichia coli* can downregulate the heat shock response by degrading the heat shock transcription factor σ^32^ [26]. The *FtsH* gene from *Bacillus subtilis* can be induced by heat and hypertonic conditions [27], while knockout of *FtsH* in this same bacterial species induces resistance to salt and is heat-stress sensitive [28]. The expression of the *FtsH* gene in wine bacteria increases under high temperature and osmotic stress and can compensate for the growth defects of *E.* *coli* FtsH mutants under heat shock conditions [29].

In tomatoes, the expression of FtsH is not only induced by heat shock, but also occurs across specific developmental stages [30]. In *Arabidopsis*, the *FtsH6* gene is expressed at very low transcription levels under normal conditions (Arabidopsis eFP browser, http://bar.utoronto.ca/efp_arabidopsis) (accessed on 27 June 2021). Under heat stress, only plastid-localized FtsH6 metalloprotease (AT5G15250) accumulated rapidly among 39 plastid protease genes, as shown in heat-induced Arabidopsis RNA-Seq data [31]. After heat stress, the tomato *LeftsH6* gene exhibited high levels of heat-induced GUS staining in the leaves, roots, and flowers of transgenic tomatoes [30]. In addition, other studies have also found that under heat stress conditions, the *FtsH6* gene was upregulated in rapeseed [32], wheat [33], and sorghum [34]. However, the heat inducibility of FtsH6 shows that nuclear evolutionary conservation in plants implies a crucial important role in response to environmental stress. No experimental evidence regarding the role of the *CaFtsH06* gene in pepper abiotic stress tolerance has been reported so far. Thus, the current study was undertaken to examine the function of *CaFtsH06*.

Pepper (*Capsicum annuum* L.) is an economically important solanaceous crop that is grown worldwide for its unique taste and use in different food industries [35,36,37]. Additionally, pepper is a thermophilic vegetable, and its growth and production are sensitive to various abiotic stresses [4,38]. In this study, we elucidated the functions of *CaFtsH06* in pepper abiotic stress tolerance while also identifying the subcellular localization CaFtsH06 proteins. The transcriptional regulation of *CaFtsH06* in response to extreme temperatures, saline conditions, and drought stress was also examined. Furthermore, the function of this gene was also evaluated by silencing *CaFtsH06* in pepper and ectopically expressing it in Arabidopsis. The findings of the study indicate that, the *CaFtsH06* gene positively regulated the abiotic stress tolerance in pepper. Our results provide a basis for further studying the function of this key gene in other important crops to improve tolerance to various stresses in variable environments.

## 2. Results

### 2.1. The Expression Pattern of CaFtsH Genes in Pepper Across Different Tissues during Development and Stress Responses

**Figure 1:**
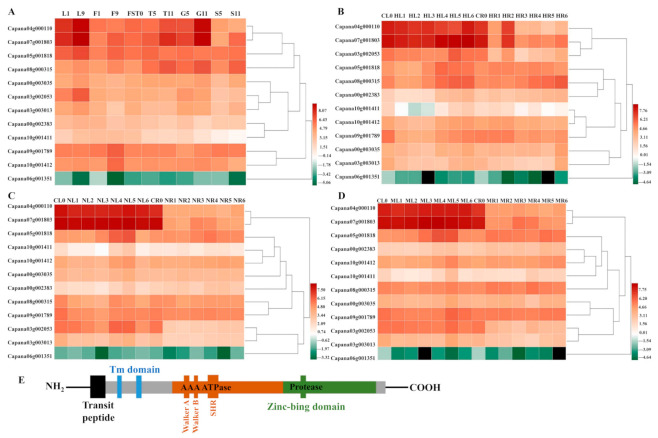
Heat map of *CaFtsHs* family gene expression in pepper. (**A**) Dynamic heat map of the *CaFtsHs* family genes in different tissues and organs of pepper. L1 and L9 stand for leaves collected at 2 d and 60 d after emergence, respectively; F1 and F9 describe the smallest and largest flower buds, respectively; FST0 indicates the fruit collected at 3 days after flowering (DAF); T5 and T11 stand for placenta collected at 30 and 60 (DAF), respectively; S5 and S11 show seeds collected at 30 and 60 DAF, respectively; G5 and G11 represent peels collected at 30 and 60 DAF, respectively. (**B**) Expression pattern of *CaFtsHs* family genes in pepper under heat stress. CL0/CR0 stand for control in leaves/roots, respectively, and HL/HR stand for expression levels of *CaFtsHs* genes after heat stress (42 °C) in leaves/roots at 0, 0.5, 1, 3, 6, 12, and 24 h t, respectively. (**C**) Expression of *CaFtsHs* family genes in pepper under salt stress (200 mM NaCl). CL0/CR0 represent control in leaves/roots, respectively; NL/NR stand for levels of *CaFtsHs* genes in leaves/roots at 0, 0.5, 1, 3, 6, 12, and 24 h post-treatment, respectively. (**D**) Profile of CaFtsHs family genes in pepper leaf and root tissues under osmotic stress (400 mM mannitol). CL0/CR0, ML1/MR1, ML2/MR2, ML3/MR3, ML4/MR4, ML5/MR5, and ML6/MR6 show the expression levels of *CaFtsHs* genes in leaves/roots at 0, 0.5, 1, 3, 6, 12, and 24 h post-treatment, respectively. The date was normalized with log2 transformation. (**E**) The conserve motifs of *CaFtsH* are shown in different colors. TM (transmembrane domain), SRH (second region of homology), Zn^2+^ metalloprotease domain (protease), and the conserved ATPase domains of Walker A and B are marked. Two transmembrane domains (TM domain, blue) and transit peptide (black) regions are shown.

The transcription data set of the 12 *CaFtsHs* genes were obtained from the pepper transcriptome database, with transcription data for the *CaFtsHs* genes at 11 different developmental stages (including six tissues). These data were drawn into a heat map with performing hierarchical clustering (Figure 1A). The heat map profile showed that *CaFtsH06*, *CaFtsH01* and *CaFstH05* genes were significantly upregulated. Among them, the *CaFtsH06* was expressed in leaves (L1, L9) and flowers (F9). *CaFstH05* (S5) was clearly expressed in seeds (S5), *CaFtsH01* was expressed at higher levels in fruits (FST0), while the expression of *CaFstH04* was highest in seeds (S11). *CaFstH09* and *CaFstH10* were only expressed at higher levels in flowers (F9), and *CaFstH11* also showed higher expression levels in leaves (L9). The expression levels of *CaFstH02* and *CaFstH08* were not high in all the tested pepper tissues. In addition, the *CaFstH12* gene was almost undetectable in all the tested tissues.

The dynamic expression patterns of *CaFtsH* gene family members under different stress treatments (osmotic, heat, and salt stress) were also observed (Figure 1B–D). The expression level of *CaFtsH06* gene in pepper leaves gradually increased after 6 h of continuous heat shock and was quite distinct at 24 h (Figure 1B). However, the expression levels of *CaFtsH06* and *CaFtsH01* first decreased after heat treatment, slightly increased after 1 h, and again decreased rapidly in the root tissue of pepper plants. After salt stress treatment, the *CaFtsH06* gene was mainly accumulated in the leaves and its accumulation at 12 h was highest (Figure 1C). By observing the expression characteristics of *CaFtsH* homologs under 400 mM mannitol stress (osmotic stress) at different periods (0, 0.5, 1, 3, 6, 12, and 24 h), it was found that the expression of *CaFtsH06* in pepper leaves under osmotic stress was most significant at 6 h of treatment (Figure 1D). After osmotic stress, *CaFstH01*, *CaFstH02*, *CaFstH05*, *CaFstH06*, *CaFstH09*, and *CaFstH10* were upregulated in pepper leaves, while *CaFstH02*, *CaFstH04*, *CaFstH05*, *CaFstH07*, *CaFstH08*, *CaFstH09*, and *CaFstH10* were upregulated in the roots. Under the stress of different periods of heat, salt, and osmotic stresses, the *CaFstH06* gene had the strongest response in pepper leaves, while the *CaFstH12* gene maintained a very low expression level in both pepper leaves and root tissue. The above results indicated that *CaFtsH06* may be involved in a variety of environmental stress response processes. Moreover, most of the other genes belonging to the family were not significantly altered in terms of expression across multiple stress conditions (Figure 1). Gene structure and function are closely related, and the homologous *LeFtsH6* and *AtFtsH6* genes were noted in other reports to have had similar stress responses [30,31]. Therefore, *CaFtsH06* was selected as the focus for further research.

FtsH is a typical membrane-bound AAA protease family member39. The structure of FtsH protease in pepper is the same as that of other biological FtsH homologs. It is characterized by a N-terminal region with one or two transmembrane (TM) helix domains required for oligomerization and a central AAA ATPase module. Additionally, the C-terminal domain of the conserved zinc binding site (HEXXH) is required for proteolytic activity [22]. By combining previous studies and the determination of the protein structure from a prediction website, Figure 1E and Figure 3 provides a schematic diagram of FtsH genes, describing the hypothetical structure of FtsH protease in Arabidopsis and pepper.

### 2.2. Expression of CaFtsH06 under Heat Stress in Pepper

**Figure 2:**
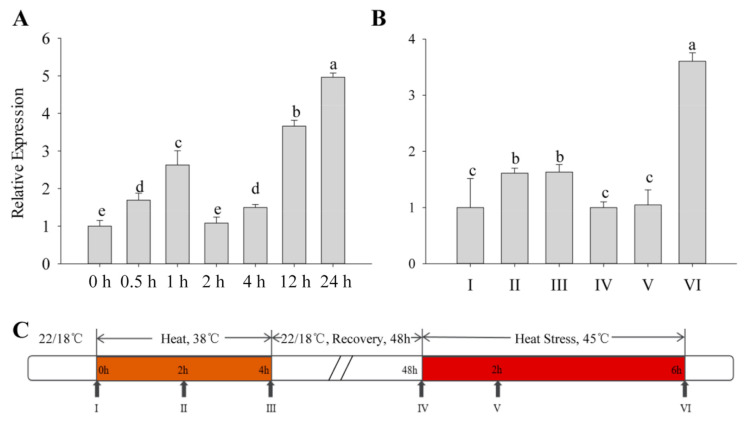
Analysis of the change in *CaFtsH06* expression in pepper plants under heat stress. (**A**) Detection of background heat tolerance of *CaFtsH06* in pepper. (**B**) Detection of acquired heat tolerance associated with *CaFtsH06* in pepper. (**C**) A time course of high temperature treatment and normal temperature recovery, with the arrows indicating the time points at which pepper leaves were collected (samples I–VI). Different letters denote statistical significance (*p* < 0.05).

All types of exogenous heat stress from the outside acts on plants, and the severity of their damage mainly depends on how fast the plant responds to the stress. The expression patterns of the *CaFtsH06* gene in R9 heat-tolerant pepper lines that were exposed to different heat treatments were determined. First, the basic thermotolerance treatment of R9 was analyzed (Figure 2A). At the beginning of the treatment, the expression of *CaFtsH06* in pepper increased slowly and then decreased after 2 h of treatment, but after 24 h, the expression of *CaFtsH06* increased rapidly to five times the expression level that occurred after 2 h of treatment. The acquired thermotolerance treatment of R9 plants (Figure 2B) between hours 2 and 4 of the heat acclimation was also analyzed. The expression level of *CaFtsH06* in leaves was not obvious and tended to be flat. In the recovery phase at 22 °C, the expression level of *CaFtsH06* first appeared to decrease, with an expression level that was similar to that level before treatment. However, after the high-temperature treatment at 45 °C, the expression level of *CaFtsH06* rose rapidly after treatment and reached its maximum at 6 h, and its expression level was more than 3 times that of the control plant (38 °C, 4 h), as Figure 2C shows, for high-temperature treatment and normal temperature recovery.

### 2.3. Structure Analysis of CaFtsH06 Proteins

**Figure 3:**
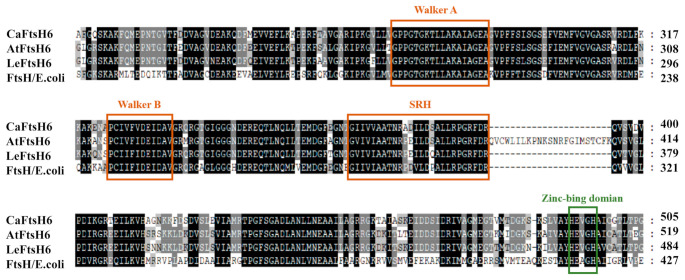
Analysis of the characteristics of FtsH protein structures. Multiple alignments of the deduced amino acid sequences of CaFtsH06, AtFtsH6, LeFtsH6, and FtsH/*E.* *coli*, with the FtsH-specific motif shown by a rectangular box. Part of the protein sequence is drawn with a colored rectangle to match the figure in (Figure 1E).

Based on the close phylogenetic relationship between tomato (*Solanum lycopersicum* L.) and pepper plants, we determined that the gene should be designated as *CaFtsH06* (GenBank accession number XP016580880) based on its homology to tomato metalloproteinase LeFtsH6 (NP001234191). The full-length *CaFtsH06* cDNA is 2301 bp, with a 194-bp 5′ untranslated region (5′UTR), 70-bp 3′untranslated region (3′UTR), and 2037-bp ORF. The corresponding protein molecular weight is 77.54 KDa, isoelectric point is 6.29 and instability index is 31.99 (Supplementary Table S1). Multiple alignments of the deduced amino acid sequences of CaFtsH06, AtFtsH6, LeFtsH6, and FtsH/*E.* *coli* revealed a consensus region (Figure 3). The central region of the CaFtsH06 contains two putative ATP binding sites (Walker A, Walker-B) and a putative SRH (the second region) for homology. The C-terminus of the CaFtsH06 protein contains a putative zinc-binding motif HEXGH (where each X is a non-conserved amino acid residue).

### 2.4. Subcellular Localization of CaFtsH06 Proteins

**Figure 4:**
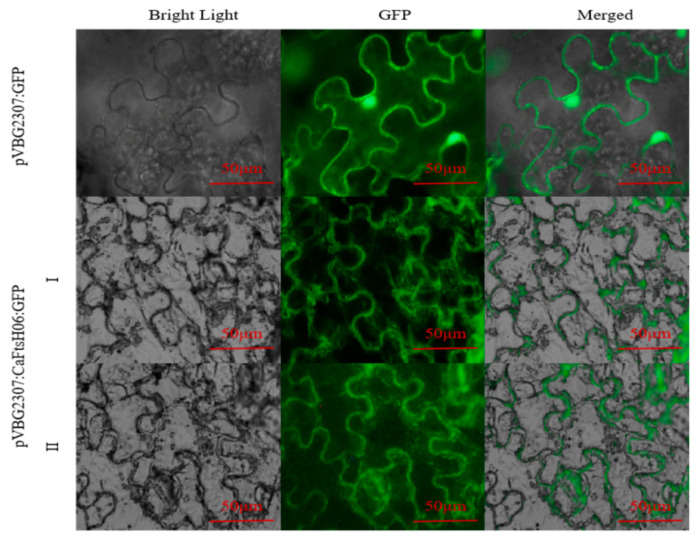
Analysis of the characteristics of CaFtsH06 protein’s subcellular localization. The localization of pVBG2307:CaFtsH6:GFP fusion protein in tobacco cells (I-II), with pVBG2307:GFP as a control. Bright light: cells in bright field; GFP: fluorescence of GFP protein under green fluorescence; merged: overlapped image of GFP and bright light. Bar = 50 μm.

The subcellular localization of CaFtsH06 was predicted using the online tool WOLF PSORT (https://wolfpsort.hgc.jp/) (accessed on 10 August 2020), which predicted that CaFtsH06 localized mainly to the mitochondria and cell membrane. To confirm the subcellular localization of CaFtsH06 protein, the pVBG2307:GFP and pVBG2307:CaFtsH06:GFP fusion plasmids were transiently expressed in tobacco leaves. We found that the green fluorescence signal of pVBG2307:CaFtsH06:GFP was detected in the tobacco leaf mitochondria and cell membrane, while the fluorescence of pVBG2307:GFP was distributed throughout the cell, indicating that CaFtsH06 protein in pepper may play a role in the mitochondria and cell membrane (Figure 4).

### 2.5. Knockdown of CaFtsH06 Decreased Abiotic Stress Tolerance in Pepper

**Figure 5:**
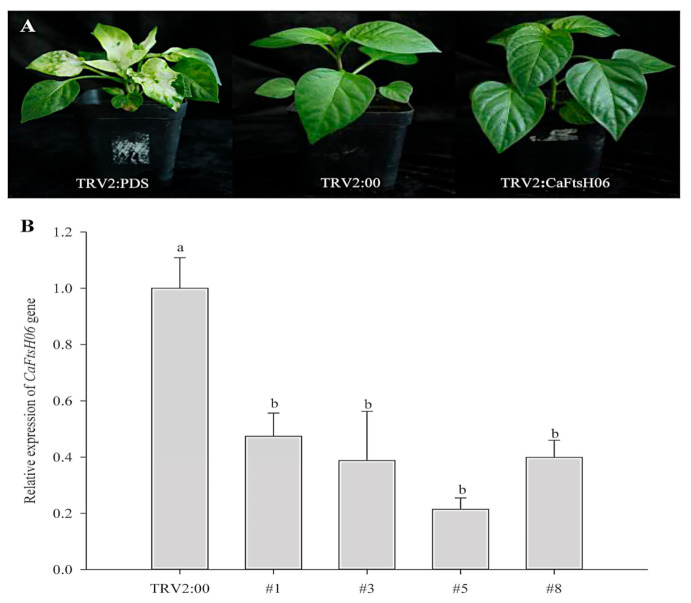
The phenotypes and silencing efficiency of *CaFtsH06* in pepper leaves. (**A**) The phenotypes of TRV2:*CaPDS* positive control plants, TRV2:00 negative control plants, and pTRV2:*CaFtsH06*-silenced plants are shown, respectively. (**B**) Silencing efficiency of *CaFtsH06* in pepper. Different letters denote statistical significance (*p* < 0.05).

The silencing vector TRV2: *CaFtsH06* was constructed in the *Agrobacterium* GV3101 to create *CaFtsH06*-silenced plants. The leaves of TRV2:*CaPDS* (positive control) pepper seedlings showed photobleaching symptoms, but no obvious difference in phenotype was observed between TRV2:*CaFtsH06* and the negative control TRV2:00 pepper lines under normal growth conditions (Figure 5A). The silencing efficiency of *CaFtsH06* in silenced pepper lines exceeded 60% (Figure 5B). Thus, the control plants (TRV2:00) and *CaFtsH06*-silenced plants (TRV2:*CaFtsH06*) were used for the subsequent experiment.

**Figure 6:**
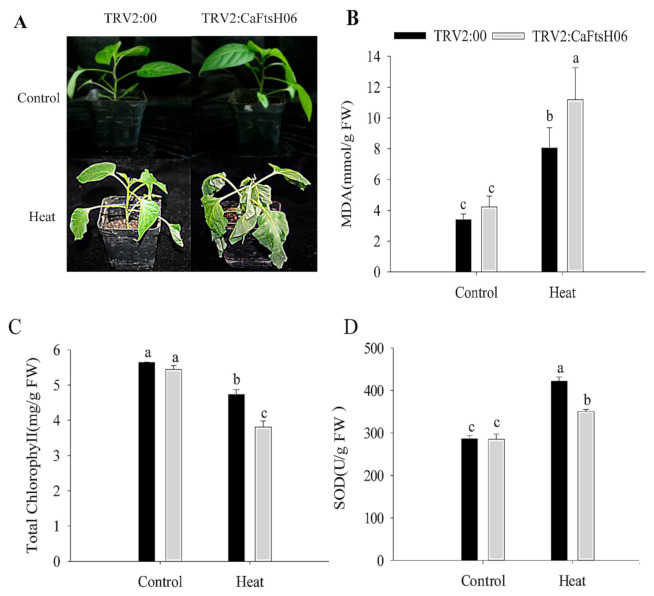
Analysis of heat tolerance of *CaFtsH06*-silenced pepper. (**A**) Phenotype identification of pTRV2:00 and pTRV2:*CaFtsH06*-silenced plants after heat treatment. (**B**) The malondialdehyde (MDA) content of plants after heat stress. (**C**) Total chlorophyll content of plants after heat stress. (**D**) The SOD content of plants after heat stress. Different letters denote statistical significance (*p* < 0.05).

The *CaFtsH06*-silenced and control R9 pepper plants were exposed to 45 °C for 24 h, and significant differences in their phenotypes were observed after high temperature treatment. The leaves of the silenced plants showed severe wilting and drooping, while control plants only exhibited slightly curled edges of leaves (Figure 6A). At the same time, it was determined that the malondialdehyde (MDA) content of the silenced and control plants increased after heat stress, but this increase in the MDA content of the silenced plants was significantly higher than that in control plants (Figure 6B). Similarly, the chlorophyll content of both silenced and control plants decreased after heat shock, but this decrease in the chlorophyll content of the silenced plants was significantly lower than that in control plants (Figure 6C). After the heat stress stimulation, the SOD content of the control and silenced plants was increased, but the increase in the SOD content of the silenced plants were lower than that of the control plants (Figure 6D). The analysis of the above results showed that silencing of the *CaFtsH06* gene in pepper reduces its ability to respond to heat shock.

**Figure 7:**
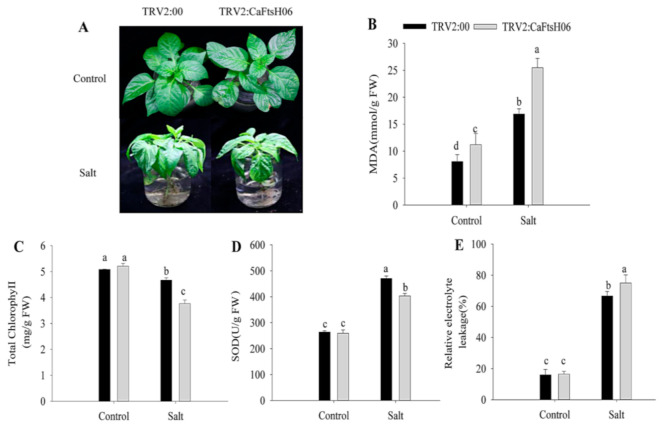
Salt stress tolerance analysis in silenced pepper. (**A**) Phenotypes of pTRV2:00 and pTRV2:*CaFtsH06*-silenced plants after salt stress. (**B**) The MDA content of plants after salt stress. (**C**) Total chlorophyll content of plants after salt stress. (**D**) The SOD content of plants after salt stress. (**E**) The relative electrolyte leakage of plants after salt stress. Different letters denote statistical significance (*p* < 0.05). For the osmotic tolerance analysis of silent and control plants, the roots of the plant samples were cleaned first and then completely immersed in a 300 mM mannitol solution. The silent and control plants were then subjected to 36 h of drought treatment, and the leaves of *CaFtsH06*-silenced plants were observed to exhibited lost water tension, sagging and shrinking, while the control plants did not change significantly (Figure 8A).

To further explore the function of CaFtsH06 in saline conditions, the CaFtsH06-knockdown and control pepper seedlings were immersed in a 300 mM NaCl solution for 24 h. The leaves of the CaFtsH06-silenced plants showed severe wilting and shrinkage, with the lower leaves having fallen off, while the leaves of the control plants only showed signs of wilting (Figure 7A). At the same time, the MDA content and REL of the two plant types increased significantly, but the increase in the silenced plants was statistically more than that in the non-silenced pepper plants (Figure 7B,E). Additionally, the chlorophyll content of the CaFtsH06-silenced and control plants decreased, but this decrease was significantly lower in the silenced plants than in the control plants (Figure 7C). The SOD content of the control and silenced plants increased after salts stress, but this increase in the SOD content of the control plants was significantly higher than that in the silenced plants (Figure 7D).

**Figure 8:**
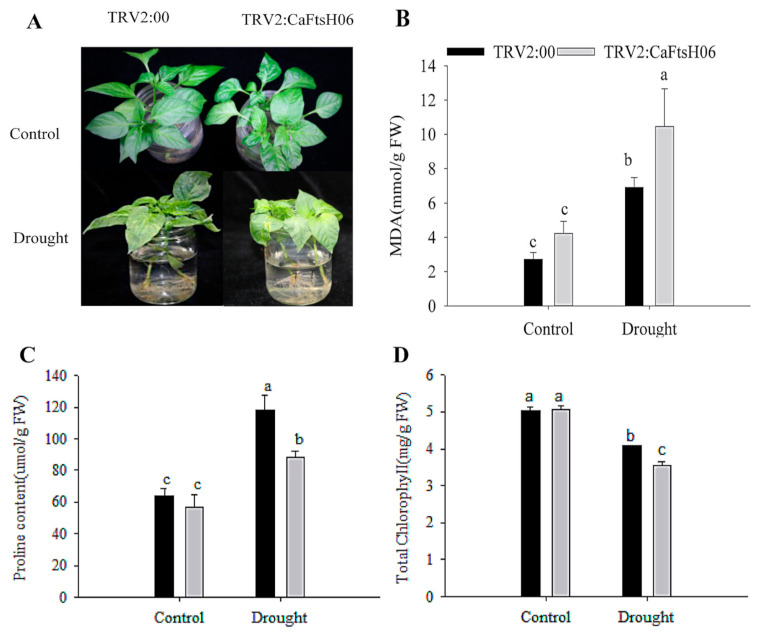
Analysis of osmotic tolerance of *CaFtsH06*-silenced pepper. (**A**) Phenotype identification of pTRV2:00 and pTRV2:*CaFtsH6* plants after osmotic stress. (**B**) The MDA content of plants after osmotic stress. (**C**) The proline content of plants after osmotic stress. (**D**) Total chlorophyll content of plants after osmotic stress. Different letters denote statistical significance (*p* < 0.05).

After treatment, the MDA content of the *CaFtsH06*-silenced plants increased significantly, more than that of the control plants (Figure 8B). Plant leaves initiate stress responses to external stimuli and rapidly accumulate proline, but the accumulation of proline in the *CaFtsH06*-silenced plant leaves was not increased (Figure 8C). Similarly, the chlorophyll content of the *CaFtsH06*-silenced plants was significantly reduced as compared to the control plants (Figure 8D). Thus, it was preliminarily determined that silencing the *CaFtsH06* gene in pepper reduces osmotic tolerance in pepper.

Osmotic stress can lead to the disordering of ion distribution and balance in plants, the deposition of large amounts of destructive ROS substances, and damage to the internal structures and the function of cells. Thus, it was important to explore the influence of *CaFtsH06* silencing on the accumulation and removal of ROS in the pepper R9 lines. Accordingly, we used histochemical staining DAB and NBT methods to measure the changes in H_2_O_2_ and ·O_2_^−^, respectively.

**Figure 9:**
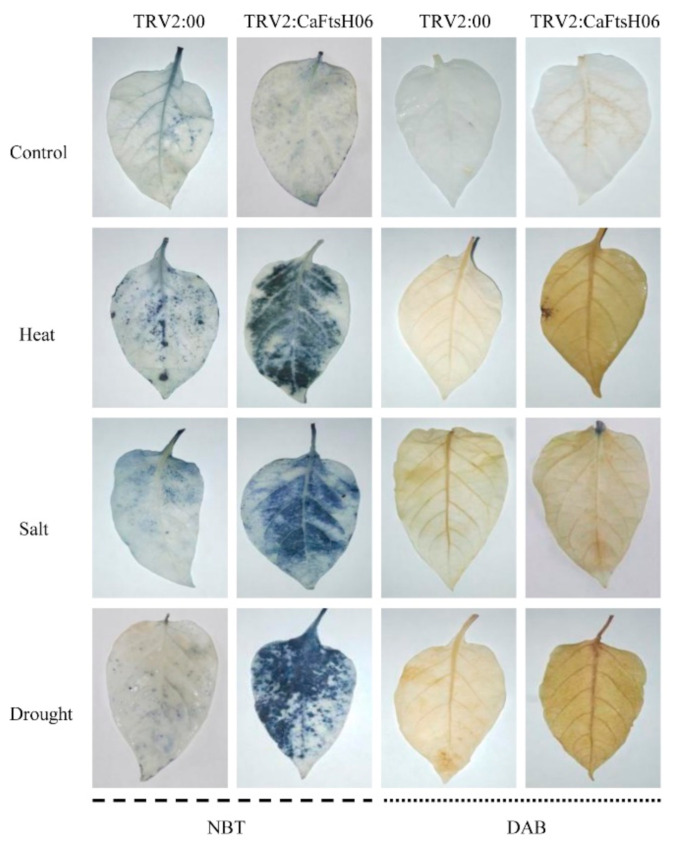
Histochemical staining of pepper leaves under heat, salt, and osmotic stresses in the *CaFtsH06*-silenced and control pepper plants. The NBT and DAB staining in *CaFtsH06*-silenced plant leaves after heat, salt, and drought stress treatments revealed darker stanning patterns than the control plants, indicating the accumulation of ·O_2_^−^ and H_2_O_2_, respectively.

As shown in Figure 9, the DAB deposition area of the *CaFtsH06*-silenced and control plants was higher after stressed conditions as compared to the unstressed plants, but the stained areas of the *CaFtsH06*-silenced plants were significantly larger than those of the control plants. Similarly, after coercion treatment, the NBT deposition areas of silenced and control peppers were significantly larger than those of unstressed plants, while the stained areas of *CaFtsH06*-silenced plants were significantly larger than those of control plants. These results indicate that as compared to the leaves of control plants, the leaves of *CaFtsH06*-silenced plants had higher levels of H_2_O_2_ and ·O_2_^−^. Thus, when the *CaFtsH06* gene was silenced in pepper, the ability of the plants to remove harmful substances such as peroxides was also reduced, such that the ability of pepper to resist stress was greatly reduced.

### 2.6. Ectopic Expression of CaFtsH06 Enhances the Abiotic Stress Tolerance in Arabidopsis

In order to determine whether the *CaFtsH06* gene was integrated into Arabidopsis, we first verified Arabidopsis at the DNA level. Based on the performance of this gene in silenced pepper plants under different abiotic stresses and the presence of multiple stress-related cis elements in the promoter region, it was speculated that overexpression of this gene in *Arabidopsis* can enhance tolerance to multiple abiotic stresses. After PCR detection, we observed that the OE7 and OE9 strains expressed *CaFtsH06* genes at a much higher levels than observed in untransformed wild-type (WT) Arabidopsis (Supplementary Figure S3B). Furthermore, we found that the *CaFtsH06* gene expression in the two lines of OE7 and OE9 under normal growth conditions was higher and that the leaf area of the transgenic lines was significantly larger than that of WT plants (Figure S4), in addition to their more vigorous phenotype after heat-stress treatment at 45 °C for 16 h. Thus, we selected these two transgenic lines (OE7 and OE9) for the following experiments.

**Figure 10:**
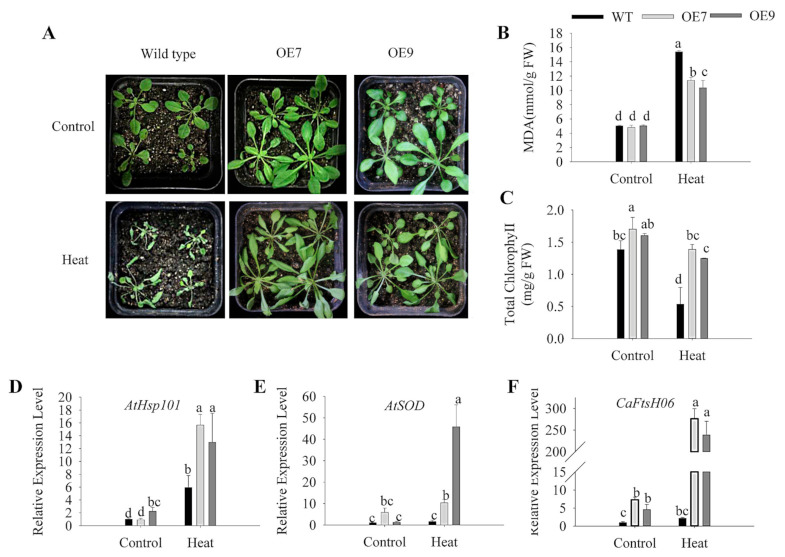
Heat stress tolerance analysis of WT and *CaFtsH06*-expressing Arabidopsis lines following 40 °C treatment for 24 h. (**A**) The phenotype of WT and *CaFtsH06*-expressing Arabidopsis lines. (**B**,**C**) MDA and total chlorophyll contents of activity of WT and transgenic Arabidopsis. (**D**–**F**) Relative expression level of WT and *CaFtsH06*-expressing Arabidopsis lines. Different letters denote statistical significance (*p* < 0.05).

To study the effect of *CaFtsH06* on the temperature tolerance of transgenic Arabidopsis, three-week-old WT and transgenic plants with consistent growth were selected and heat treated at 40 °C for 24 h. Phenotypic observation (Figure 10A) revealed that the WT leaves exhibited dryness and wilting, with necrosis on some leaves. In contrast, the *CaFtsH06*-expressing transgenic line only showed curling and wilting on the leaf edges, and its phenotype was obviously more vigorous than that of WT plants. At the same time, leaves from WT plants and the *CaFtsH06*-expressing transgenic line were collected before and after treatment for the determination of their physiological indicators related to stress resistance. Analysis of MDA content (Figure 10B) revealed that high-temperature treatment significantly increased the MDA content of OE7, OE9, and WT leaves, though the MDA content of the *CaFtsH06*-expressing lines were significantly lower than that of WT plants. After heat treatment, the chlorophyll content of the WT leaves decreased significantly (Figure 10C). Additionally, the expression levels of the heat-stress response gene (*AtHSP101*) and SOD enzyme synthesis gene (*AtSOD*) in OE7 and OE9 plants were significantly higher than those of WT plants (Figure 10D,E), and at the same time, the expression of *CaFtsH06* in OE9 and OE7 was also increased significantly (Figure 10F).

**Figure 11:**
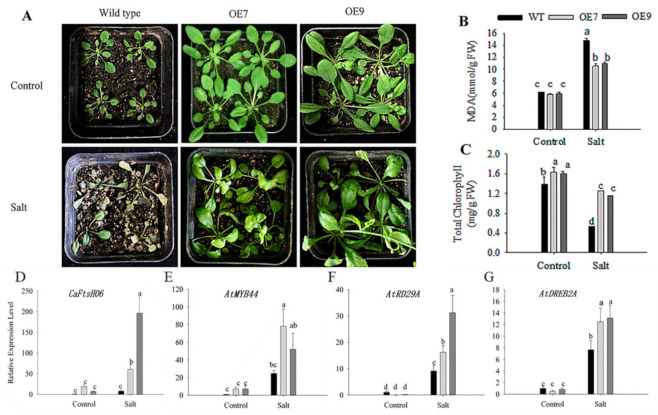
Phenotypic observations and physiological and transcriptional analysis after salt stress in the WT and *CaFtsH06*-expressing Arabidopsis plants watered with a 200 mM NaCl solution for 14 days. (**A**) Phenotypes of *CaFtsH06*-OE Arabidopsis and WT plants. (**B**,**C**) MDA and chlorophyll contents of the WT and transgenic Arabidopsis plants. (**D**–**G**) Expression levels of *CaFtsH06* and other stress-related genes in transgenic Arabidopsis and WT plants under salt stress. Different letters denote statistical significance (*p* < 0.05).

The damage induced by salt stress to plants also involves a series of physiological and biochemical stress responses, which can cause obvious phenotypic damage in severe cases. The WT and transgenic line seedlings at the age of three weeks were watered with a 200 mM NaCl solution once every two days for two weeks. As compared with the control plants, the WT plants showed severe chlorosis and wilting of their leaves after salt stress treatment. However, the transgenic plants showed no obvious difference from the control plants in other characteristics, except for the slight yellowing phenotype in their leaves (Figure 11A). At the same time, the leaves of WT, OE7, and OE9 plants were collected for the determination of their MDA and chlorophyll contents before and after the salt stress treatment. After salt treatment, the MDA content of WT, OE7, and OE9 leaves significantly increased, but the increase in MDA content of WT plants was higher than that of OE7 and OE9 plants (Figure 11B). The chlorophyll content in Arabidopsis leaves was also significantly reduced after the stress treatment, but the chlorophyll content in WT leaves was significantly lower than that of the OE7 and OE9 lines (Figure 11C). To further study the salt tolerance mechanism, qRT-PCR was used to analyze changes in the expression of salt-stress related genes in the WT and OE lines before and after salt treatment. The expression of *CaFtsH06* (Figure 11D) and the salt-stress-responsive genes *AtMYB44*, *AtRD29A*, and *AtDREB2A* increased significantly in the OE7 and OE9 lines (Figure 11E–G) as compared to the WT plants. Thus, ectopic expression of the *CaFtsH06* gene in Arabidopsis was beneficial to alleviate the salt damage to Arabidopsis plants.

**Figure 12:**
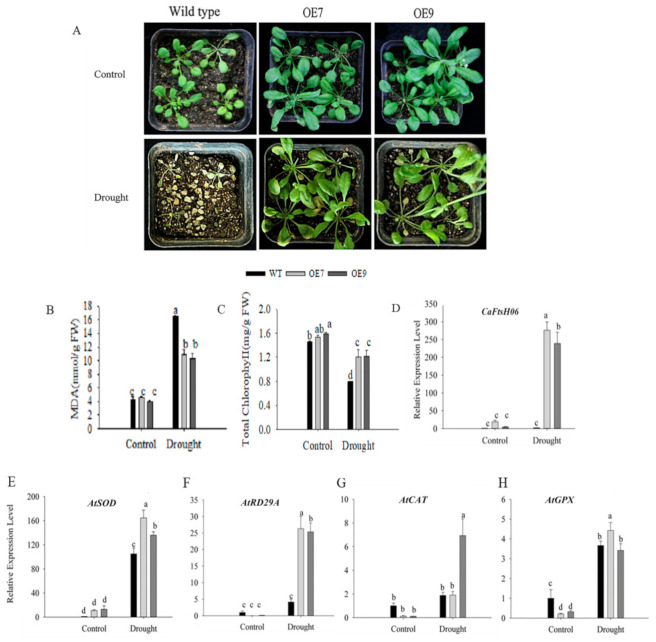
Drought stress tolerance analysis of wild-type (WT) and transgenic CaFtsH06-expressing Arabidopsis seedlings. (**A**) Phenotypes of WT and CaFtsH06-expressing Arabidopsis seedlings following the withholding of water for seven days. (**B**,**C**) MDA content and total chlorophyll content of CaFtsH06-expressing and WT Arabidopsis plants. The drought stress was applied by not watering the seedlings for seven days. (**D**–**H**) The transcript levels of genes related to drought stress in the transgenic and WT Arabidopsis plants. Different letters denote statistical significance (*p* < 0.05).

Drought stress, like other stress conditions, also induces a series of morphological, physiological, biochemical, and molecular stress changes in plants. For the drought tolerance assay, the WT and transgenic seedlings (three weeks old) were selected for water-control and drought treatment. After the drought treatment, the leaves of WT seedlings were severely wilted and necrotic, while some of the leaves of the OE7 and OE9 lines appeared dry and yellow (Figure 12A). In addition, the MDA content of WT and transgenic plants increased after drought treatment, but the MDA content of the transgenic plants was significantly lower than that of WT plants (Figure 12B). The chlorophyll content of the WT plants decreased significantly after treatment, though that of the transgenic plants was higher than the WT plants (Figure 12C). Subsequently, we measured the expression levels of the drought-stress response genes and *AtRD29A*, *AtCAT*, *AtGPX*, and the SOD enzyme synthesis gene *AtSOD* in leaves. The expressions of the *CaFtsH06* and the four drought-stress related genes in the transgenic plants were all upregulated after the drought treatment, and each was significantly higher than those of WT plants (Figure 12D,F–H). Collectively, the ectopic expression of the *CaFtsH06* gene in Arabidopsis effectively enhanced the resistance of Arabidopsis to drought stress.

**Figure 13:**
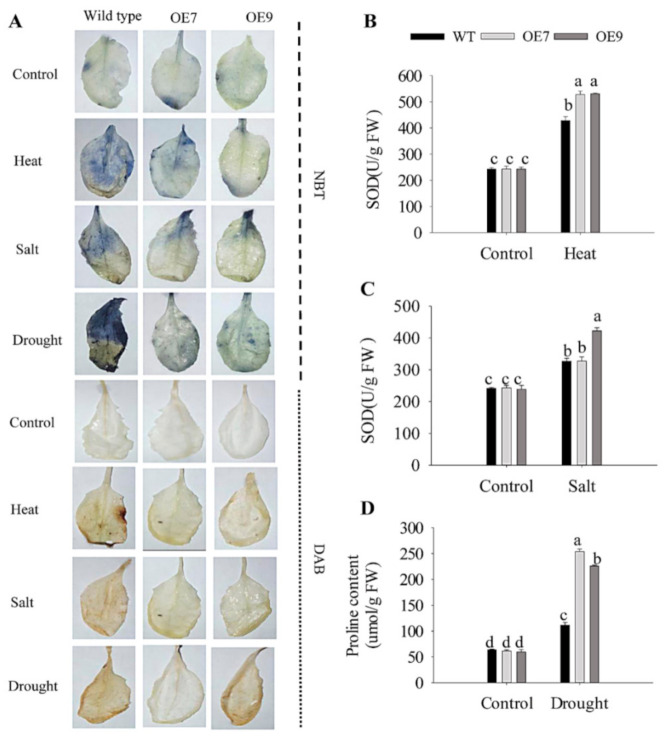
Study on the scavenging characteristics of reactive oxygen species (ROS) of wild-type and transgenic plants. (**A**) NBT and DAB staining of WT and transgenic plants after different stress treatments. (**B**) SOD activity of WT and transgenic lines under heat stress. (**C**) SOD activity of WT and transgenic plants under salt stress. (**D**) Proline content of WT and transgenic lines under drought stress. NBT and DAB staining showed accumulation of ·O_2_^−^ and H_2_O_2_, respectively. The error bars show the SD of three biological replicates, and values shown with different lowercase letters were significantly different at a *p* ≤ 0.05 level of significance. Different letters denote statistical significance (*p* < 0.05).

In the processes of plant metabolism, redox reactions occur inside and outside of cells. Based on the NBT and DAB staining of the leaves of plants after stress treatment, the ability of transgenic *CaFtsH06*-expressing Arabidopsis to resist adversity stress and eliminate harmful ROS substances was measured. Compared with the control, the DAB and NBT staining of the WT plants were stronger after the stress treatment, while the accumulation of peroxides in the leaves of the transgenic plants was distinctly lower. For plants not subjected to stress treatment, the stained areas of leaves of the WT plants were significantly higher than those of transgenic plants (Figure 13A). Under heat stress treatment, compared with the wild-type Arabidopsis, the SOD activity of the transgenic lines was higher (Figure 13B). After the same salt treatment, the SOD content of the transgenic Arabidopsis leaves was also higher than that of the wild-type Arabidopsis. The SOD content in the leaves of the WT plants was high (Figure 13C). The proline content in the leaves of the transgenic and WT plants was also measured. The transgenic plants responded quickly to drought stress, with a more obvious increase in the proline content of leaves (Figure 13D). Altogether, the ectopic expression of the *CaFtsH06* gene in Arabidopsis improved the ability of plants to resist stress and accelerate the elimination of harmful ROS.

## 3. Discussion

When plants encounter harsh environments such as extreme temperatures, drought, and high salinity, the resulting oxidative stresses cause disordering of their protein functions to occur. Many proteins lose their functions, owing to denaturation, and then condense together to form aggregates that affect the proteins in the plant or gene functions, which can cause serious damage to all aspects of plants [38,39,40]. FtsH not only participates in repairing damaged proteins, unfolded proteins, and unassembled free subunits, but also can control heat response and lysogen by interacting with some regulatory factors (such as σ^32^, λCII, LpxC) and other cell activities [11,41,42]. Additionally, FtsH is involved in the EXECUTER1 (EX1)-mediated retrograde signaling network induced by singlet oxygen (^1^O_2_) [43]. Recent research by Dogra, et al. [44] claimed that FtsH regulation prevents ^1^O_2_ signaling and the expression of related genes in flu mutants. Previous studies have shown that the expression of many FtsH proteases can be induced by abiotic stress [20,21,22,41]. Our results demonstrated that the *CaFtsH06* gene responded to abiotic stressors such as extreme temperature, drought, and saline conditions (Figure 1). The *CaFtsH06* transcript levels differed and exhibited a complex pattern across different temperature conditions. (Figure 2). Previous reports showed that the *FtsH6* gene is involved in thermotolerance in tomatoes [30]. These findings showed that, the *CaFtsH06* gene may participate in plant responses to variable environmental stimuli, such as heat, salt, and drought stress.

FtsH localized to various areas [10], such as the protease pairs FtsH3⁄FtsH10 localized to mitochondria [45], FtsH11 localized to both chloroplasts and mitochondria [17], and AtFtsH6 localized to chloroplast, where it may protect the thylakoid membrane of plants under heat stress [31]. In this study, we observed that *CaFtsH06* enters the mitochondria and cell membrane, (Figure 4), suggesting that CaFtsH06 protein may play a fundamental role in the mitochondria and cell membrane.

Under heat, drought and salt stress conditions, plant cell structures suffer while physiological, biochemical, and molecular damage may occur, further leading to cell death [1,5]. Additionally, such stress can cause an increase in the MDA content and leaf REL [46]. MDA content is an indicator of the defense signaling of cell membrane lipid peroxidation [47]. In this study, the MDA content of the *CaFtsH06*-silenced peppers was higher than that of the control (TRV2:00) peppers under drought and salt-stress conditions (Figure 6B, Figure 7B and Figure 8B). This demonstrated that silencing *CaFtsH06* increased the damage to the cell membrane in pepper. Furthermore, silencing of *CaFtsH06* in pepper reduced the total chlorophyll content as compared to control plants, which may in turn decrease the photosynthetic efficiency (Figure 6C, Figure 7C and Figure 8D). In addition, we found that the *CaFtsH06*-silenced peppers had higher leaf REL and proline content when exposed to salt and drought stress (Figure 7E and Figure 8C). Under different stress conditions, the excess accumulation of ROS, such as H_2_O_2_ and ·O_2_^−^, likely resulted in membrane damage and damage to other macro molecules, such as proteins, lipids, and the photosynthetic apparatus [48,49]. Therefore, the contents of H_2_O_2_ and ·O_2_^−^ were also indicators of the damage level to plant cells [50]. In the present study, the ROS level in the *CaFtsH06*-silenced pepper plants were substantially higher than in the control peppers under different environmental conditions (Figure 9). These results demonstrated that this critical gene was involved in abiotic stress tolerance as shown in the silenced plants after the abiotic stress treatments. To further elucidate the biological function of *CaFtsH06* in plant response to other environmental stresses, *Agrobacterium*-mediated ectopic expression *CaFtsH06* lines were examined in Arabidopsis. In this study, *CaFtsH06*-expressing Arabidopsis lines exhibited reduced plant sensitivity to extreme oxidative stress through increased recovery and reduced damage symptoms, MDA content, proline content, and accumulations of H_2_O_2_ and ·O_2_^−^, as well as increased total chlorophyll content and SOD activity compared to the WT plants (Figure 10, Figure 11, Figure 12 and Figure 13). Our results showed that ectopic expression of *CaFtsH06* promoted plant stress tolerance. In particular, compared to the WT line, the transgenic Arabidopsis lines exhibited thermostability associated with lower ROS content. Thus, these findings showed that *CaFtsH06* positively mediates the plant tolerance to high temperature, excess salt, and drought stress.

Many defense-related genes in plants may be involved in the rapid response to adversity stress and enhancement of plant resistance. For example, AtFtsH2 regulates the transcription of the stress response protein Hsp21, further protecting the photosynthetic system (PS Ⅱ) complex against heat stress [50]. In cotton and tobacco, ectopic expression of the *AtHsp101* gene increases heat tolerance in transgenic plants [51], suggesting that it may play a fundamental role in conferring heat stress resistance [52]. In *alfalfa*, another FtsH homolog is induced by cold stress or high light conditions [22]. The transcription factor *AtMYB* can regulate the ABA-mediated response of plants to salt and drought stress [53]. The salt-sensitive *AtDREB2A* gene, which can be induced by NaCl stress, can maintain the ion balance inside across the cell membrane [54]. Defense-related genes such as *AtSOD*, *AtCAT*, and the drought stress marker *AtRD29A* gene were upregulated by high temperatures and salt stress [55,56]. In this study, ectopic expression of the *CaFtsH06* gene increased the expression of stress-related genes (*AtHSP101*, *AtMYB44*, *AtDREB2A*, *AtCAT*, *AtGPX*, *AtSOD*, and *AtRD29A*) under high-temperature, salt, and drought stresses in the transgenic Arabidopsis relative to the WT plants (Figure 10, Figure 11 and Figure 12). Thus, we hypothesize that *CaFtsH06* might enhance the stress resistance of pepper plants by regulating the expression of stress-related genes, though further study is required to unravel the associated complex regulatory gene network. Interestingly, our results suggest that *CaFtsH06* under the constitutive 35S promoter is upregulated in response to different abiotic stresses, leading the basic tolerance. Furthermore, we will also construct the vector of the gene with its promoter, or using the Arabidopsis stress-induced promoter *RD29A*, and then after transformation of the recombinant fusion vector into Arabidopsis it would be interesting to further verify whether the gene has acquired tolerance.

## 4. Materials and Methods

### 4.1. Plant Materials and Growth Conditions

The R9 thermotolerant pepper line (introduced at the World-Asia Vegetable Research and Development Center, PP0042-51) and *Arabidopsis thaliana* ecotype Columbia-0 (Col-0) were provided by the College of Horticulture, Northwest A&F University, China. The growth conditions of the test materials were strictly managed in an incubator, with temperatures maintained at 22/18 °C, the light conditions were adjusted to 16 h/8 h (day/night), and a relative humidity of 65% [57]. Temperature was thereafter adjusted according to the experimental requirements detailed below.

### 4.2. Transcriptome Data Analysis of Pepper

To understand the expression patterns of pepper *CaFtsHs* family member genes in different tissues during development and various stress response periods, the transcriptomic data from pepper was used for analysis. The Pepper Hub (http://pepperhub.hzau.edu.cn/) (accessed on 15 March 2020) database was used to acquire the transcriptome data for *CaFtsHs* family members (12) from the Zunla-1 genome [58]. Tissue-specific expression patterns of different tissues at various developmental stages (Supplementary Table S2), including leaves (L1–9), flowers (F1-9), fruits (FST0), pericarp (G1–11), placenta (T3-11), and seeds (S3–11) were analyzed. Expression levels of the *CaFtsHs* genes in pepper leaves/roots under different stress treatments (Supplementary Table S3), including heat stress (42 °C), salt stress (200 mM NaCl), and osmotic stress (400 mM mannitol), were estimated at 0, 0.5, 1, 3, 6, 12, and 24 h, respectively. Using the HemI software (Available online: http://hemi.biocuckoo.org/down.php) (accessed on 27 June 2021) [59], the transcription data of *CaFtsHs* family members from pepper were rendered into an expression heat map.

### 4.3. Sequence Analysis and Identification of Conserved Domains

The open reading frame (ORF) of *CaFtsH06* was confirmed using the NCBI ORF Finder. The number of amino acids, the molecular weight, the theoretical isoelectric point (pI), and the protein instability index (>40 considered unstable) were obtained by analyzing amino acid sequences with the EXPASY PROTOPARAM (Available online: http://web.expasy.org/protparam/) (accessed on 27 June 2021) [60]. Conserved prediction domains, motifs, and subcellular localization were inferred using the InterProScan (Available online: http://www.ebi.ac.uk/interpro/) (accessed on 27 June 2021) [61], the PROSITE Scan (Available online: http://www.ebi.ac.uk/InterProScan/) (accessed on 27 June 2021) [62], and WoLF PSORT (Available online: https://wolfpsort.hgc.jp/) (accessed on 27 June 2021) [63], respectively. The ClustalW project (Kyoto University Bioinformatics Center, Uji, Japan) and GenDoc (Available online: http://www.psc.edu/biomed/gendoc/) (accessed on 27 June 2021) [64] were used for sequence alignment.4.4. RNA Extraction and qRT-PCR Analyses.

Total RNA extraction was performed using the method of Guo et al. [65], and reverse transcription was performed according to the manufacturer’s instructions of the TaKaRa reverse transcription kit (TaKaRa, Dalian, China). The samples were analyzed via qRT-PCR using the iQ5.0 Bio-Rad iCycler thermocycler (Bio-Rad, Hercules, CA, USA). All primer pairs used for qRT-PCR were designed using the NCBI Primer-BLAST website (Supplementary Table S4). The reaction mixture used in the PCR analyses was SYBR Green Supermix (Takara, Dalian, China). The ubiquitin ligase protein gene (*CaUbi3*) was used as a reference gene for pepper, and the *AtActin2* was used as a reference gene for Arabidopsis [65]. The relative expression levels of all the genes were calculated using the 2^−ΔΔCT^ method [66].

### 4.4. Subcellular Localization

A CaFtsH6 ORF without a stop codon was cloned from pepper cDNA. The *CaFtsH06* fragment obtained via PCR amplification was then cloned into pVBG2307:GFP vector, and the empty pVBG2307:GFP vector without *CaFtsH06* was used as a control. Constructs were confirmed using specific primer pairs (Supplementary Table S5); the constructed vector was transformed with *Agrobacterium tumefaciens* strain GV3101 to achieve transient expression of the recombinant fusion vector in the leaves of Nicotiana *benthamiana*.

### 4.5. Silencing of CaFtsH06 in Pepper

*CaFtsH06* expression was knocked down in pepper by using the virus-induced gene silencing (VIGS) technique. In the CDS region of the *CaFtsH06* sequence (Supplementary Table S6), fragments with higher specificity were used to design the primers to obtain amplicons that could be used for the construction of the pTRV2:*CaFtsH6* vector (Supplementary Table S7). A TRV2:00 empty vector was used as a negative control, and aTRV2:*CaPDS* (phytane desaturase gene) construct was used as a positive control. The TRV2:*CaFtsH06*-, TRV2:00-, and TRV2:*CaPDS*-constructed vectors were transformed by using A. *tumefaciens* strain GV3101 (Supplementary Figure S1). They were then infiltrated into the leaves of pepper R9 line pepper plants at the two-true-leaf stage. After 40–45 days, when most of the leaves of the positive control pepper plant appeared chlorotic, the TRV2:*CaFtsH06-* and TRV2:00-injected plants were analyzed via qRT-PCR for analysis of *CaFtsH06* expression, and the corresponding silencing efficiency was obtained [57].

### 4.6. Construction of Transgenic Arabidopsis Line Expressing CaFtsH06

The full-length of the *CaFtsH06* open reading frame (ORF) was cloned from pepper cDNA, and a specific primer pair containing the BamHI and KpnI restriction enzyme sites was used (Supplementary Table S8). Subsequently, the *CaFtsH06* fragment obtained via PCR amplification was cloned into the pVBG2307 vector (Supplementary Figure S2). The constructed vector was transformed into Arabidopsis using the A. *tumefaciens* strain GV3101 to realize the transformation of the recombinant fusion vector into Arabidopsis [67]. Transgenic plants were placed in an MS medium with kanamycin (50 mmol/L) to screen for resistance conferred by the vector. To determine the successfully transformed lines, first of all, DNA was extracted from the leaves of the three-week-old seedlings of T_3_ generation seedlings. The extraction was performed using the method of Huang, et al. [68] and subsequent PCR detection. At the same time, RNA was extracted separately, and the expression level of *CaFtsH06* was detected via qRT-PCR, thereby identifying five transgenic lines (OE6, OE7, OE8, OE9, and OE14) (Supplementary Figure S3). Three-week-old seedlings from the transgenic Arabidopsis lines (OE6, OE7, OE8, OE9, and OE14) were treated at 45 °C for 16 h, and the phenotypes were then observed. After comprehensively assessing the expression of *CaFtsH06* in transgenic Arabidopsis before and after heat stress as well as assaying the heat-resistant phenotype, two transgenic lines (OE7 and OE9) were selected.

### 4.7. Plant Abiotic Stress Treatments

The pepper seedlings from line R9 were grown in growth chambers with controlled photoperiod and temperature conditions. The seedlings were raised at 22/18 °C and with a photoperiod of 16 h/8 h (day/night) and a relative humidity of 65%, and samples were collected at the six-to-eight-leaf stage after the treatment with heat, salt, and drought stresses [57,65,69]. The basic thermotolerance treatment involved placing seedlings in a growth chamber set to 45 °C, with collection of plant leaves at 0, 0.5, 1, 2, 4, 12, and 24 h. The acquired thermotolerance treatment involved placing the seedlings in a growth chamber set to 38 °C for thermal acclimation and sampling after 0, 2, and 4 h of acclimatization. After the 4 h thermal acclimation, the seedlings were restored to 22 °C conditions for 48 h. Subsequently, the recovered pepper plants were treated again at a high temperature of 45 °C, and the leaves were sampled and collected at 0, 2, and 6 h, respectively. The time course of the thermal stress treatment is described in Figure 1C. In total, six to eight strains were respectively selected for mixed sampling, with three biological replicates. The samples were immediately frozen in liquid nitrogen and stored in a −80 °C freezer prior to RNA extraction.

To analyze the loss of function of the *CaFtsH06* plants under different stresses, the *CaFtsH06*-silenced pepper plants were generated as mentioned earlier. For heat stress, the seedlings were placed in a growth chamber set to 45 °C for 24 h. After exposure to heat, the collected pepper leaves were used for the determination of MDA, chlorophyll, and SOD content levels, as well as DAB and NBT staining. For salt stress, the roots of the seedlings were washed and soaked in a 300 mM NaCl solution for 24 h. Pepper leaves were collected for determination of MDA, chlorophyll, and SOD content levels, relative electrolytic leakage (REL), and staining of the leaves with DAB and NBT. For osmotic stress, after washing the roots of pTRV2:00 negative control plants and pTRV2:*CaFtsH06*-silenced plants, they were soaked in a mannitol solution (300 mM, treatment for 36 h). Pepper leaves were collected for determination of MDA, chlorophyll, SOD and proline content levels, as well as and DAB and NBT staining, while RNA was also extracted from the leaves.

To analyze the function of *CaFtsH06* in responding to abiotic stress, the three-week-old *CaFtsH06*-expressing and WT Arabidopsis lines were used. For heat stress, the seedlings were placed in a growth incubator set to 40 °C and incubated for 24 h. For salt stress, the three-week-old seedlings were watered with an NaCl solution (200 mM) every other day, with sampling after 14 d. For drought stress, water was withheld from the three-week-old seedlings, and sampling was conducted seven days later. Plants under normal conditions were used as the control. The leaves of plants before and after the treatment were sampled at random for the determination of the accumulation of MDA, chlorophyll, proline and SOD content levels, DAB and NBT staining, and RNA extraction across three replicates.

### 4.8. Biochemical Indices

The malondialdehyde content, superoxide dismutase (SOD) activity, and REL determination methods followed those described by Stewart and Bewley [70]. The chlorophyll content was determined following the method of Arkus [71]. Proline content was measured following the method described by Naser [72]. ROS presence was determined through histochemical staining with DAB and NBT following the method of Sekulska-Nalewajko [73].

### 4.9. Statistical Analysis

The statistical analyses were conducted using the SPSS 19.0 (IBM Corp. Armonk, NY, USA). Analysis of variance (ANOVA) was conducted, with significant differences considered at a *p* ≤ 0.05 threshold. All the analyses were conducted with three biological replicates.

## 5. Conclusions

Our findings show that *CaFtsH06* responds significantly to heat, salt, and drought stress and encodes a protein that localizes to the mitochondria and cell membrane. The silencing of *CaFtsH06* enhanced pepper sensitivity to heat, salt, and drought stress. However, the *CaFtsH06*-expressing Arabidopsis lines showed decreased sensitivity to heat, salt, and drought stress tolerance. Furthermore, our results provide important information about *CaFtsH06*, indicating that it may play a crucial role in tolerance of the above-mentioned stresses by inhibiting the accumulation of H_2_O_2_ and inducing the transcriptional levels of defense-related genes. Consequently, *CaFtsH06* appears to play a pivotal role in plant adaptation to environmental stimuli, and additional work should focus on the adaptive mechanisms underlying the interaction between the *CaFtsH* family genes and plant development in extreme environmental conditions.

## Data Availability

Data is contained within the article.

## Supplementary Materials

The following are available online at https://www.mdpi.com/article/10.3390/ijms22136953/s1.

## Abbreviations

ROS	reactive oxygen species
PDS	phytoene desaturase
SOD	superoxide dismutase
NBT	nitro-blue tetrazolium
DAB	diaminobenzidine
H_2_O_2_	hydrogen peroxide
·O_2_^−^	superoxide
MDA	malondialdehyde
mg/g	milligram per liter
OE	overexpression
OE#7	No. 7 *Arabidopsis* line with expressing *CaFtsH06*
OE#9	No. 9 *Arabidopsis* line with expressing *CaFtsH06*
UBI	ubiquitin-conjugating protein
ORF	open reading frame
TRV	tobacco rattle virus
VIGS	virus-induced gene silencing
