# Supplementary material for: CaFtsH06, A Novel Filamentous Thermosensitive Protease Gene, Is Involved in Heat, Salt, and Drought Stress Tolerance of Pepper (Capsicum annuum L.)
Source: Int J Mol Sci. 2021 Jun 28;22(13):6953. doi: 10.3390/ijms22136953 (PMC8268771; doi:10.3390/ijms22136953)
Supplement: Supplementary file 1 [file ijms-22-06953-s001.zip › ijms-1245452-supplementary.pdf]

Supplementary Material

Table S1. List of *CaFtsHs* family genes identified in pepper and their sequence characteristics. The proteomic information was obtained from EXPASY (Available online: http://web.expasy.org/protparam/).

Number	Name	Gene ID	Chromosome	Number of Intron	Predicted Protein Length	Molecular Weight (KD)	Isoelectric Point	Instability Index
1	CaFstH1	*Capana04g000110*	4	4	710	76.29	6.00	34.95
2	CaFstH06	*Capana07g001803*	7	3	721	77.54	6.29	31.99
3	CaFstH5	*Capana05g001818*	5	6	721	77.71	9.21	44.65
4	CaFstH4	*Capana08g000315*	8	4	422	47.31	6.49	42.19
5	CaFstH7	*Capana00g003035*	un	12	711	76.77	5.97	40.01
6	CaFstH2	*Capana00g002383*	un	5	692	77.09	7.63	34.24
7	CaFstH8	*Capana10g001411*	10	5	643	71.82	5.68	33.09
8	CaFstH9	*Capana09g001789*	9	7	820	90.47	8.39	37.14
9	CaFstH10	*Capana10g001412*	10	7	814	89.49	7.98	35.86
10	CaFstH11	*Capana03g002053*	3	16	405	44.65	6.29	45.45
11	CaFstH3	*Capana03g003013*	3	17	452	51.01	4.97	46.07
12	CaFstH12	*Capana06g001351*	6	18	480	55.36	5.40	36.37

Table S2. Transcriptome data analysis of *CaFtsHs* family genes in pepper during different tissues development.

GenID	Different tissues									
	L1	L9	F1	F9	FST0	T5	T11	G5	G11	S5	S11
Capana04g000110	286.33	710.58	76.01	63.03	96.75	83.94	160.93	148.43	641.63	31.34	22.46
Capana07g001803	336.21	838.44	53.10	122.26	92.43	124.04	306.94	202.53	755.50	26.44	94.86
Capana05g001818	116.61	178.78	59.29	105.29	77.36	102.68	70.32	84.13	69.05	69.70	76.89
Capana08g000315	31.99	43.70	32.62	51.04	36.30	82.79	137.55	89.20	142.06	46.80	117.04
Capana00g003035	24.66	36.64	20.63	16.29	23.69	27.75	28.15	26.32	33.17	12.28	25.54
Capana00g002383	13.03	17.08	15.18	8.65	11.95	8.73	6.65	9.28	4.76	7.38	7.26
Capana10g001411	5.97	10.20	11.60	10.97	9.88	4.74	4.22	5.30	2.82	1.70	1.24
Capana09g001789	62.70	58.57	71.85	118.29	53.59	56.19	54.93	35.12	50.94	67.42	60.54
Capana10g001412	39.12	42.69	35.14	100.19	39.89	42.91	39.90	42.45	46.92	36.91	21.81
Capana03g002053	59.10	132.34	24.03	18.71	25.03	19.99	18.76	38.73	29.93	8.69	11.22
Capana03g003013	26.19	36.01	18.92	7.61	20.77	12.56	12.80	27.84	24.27	8.78	17.05
Capana06g001351	0.21	0.04	0.37	0.03	0.11	0.13	0.10	0.12	0.21	0.22	0.04

Table S3. Transcriptome data analysis of *CaFtsHs* family genes in pepper during different stress treatments (including heat, salt and osmotic stress).

GenID	Leaf							Root						
	CL0	HL1	HL2	HL3	HL4	HL5	HL6	CR0	HR1	HR2	HR3	HR4	HR5	HR6
Capana04g000110	318.17	275.51	216.52	241.03	179.11	137.19	338.77	214.31	28.35	174.37	13.33	13.62	7.56	13.56
Capana07g001803	424.00	330.98	277.39	295.8	515.21	546.42	633.80	372.34	34.21	226.23	37.04	49.2	41.57	63.05
Capana05g001818	34.36	22.78	19.36	40.33	85.56	70.33	62.84	38.04	48.50	49.21	41.92	48.80	44.01	34.23
Capana08g000315	47.43	26.86	27.94	43.93	76.47	98.42	98.53	40.4	45.64	39.58	48.98	71.64	80.41	110.85
Capana00g003035	17.77	14.91	10.65	8.61	18.00	16.93	24.13	13.68	16.09	13.38	14.02	15.34	10.41	29.94
Capana00g002383	5.87	3.38	3.48	4.08	10.04	12.49	15.00	7.47	9.85	7.64	9.89	10.75	13.42	15.18
Capana10g001411	5.14	0.95	0.47	0.60	2.34	5.30	6.94	3.68	4.37	3.61	1.51	2.75	1.09	1.54
Capana09g001789	69.67	23.64	18.05	19.55	33.14	40.48	50.1	54.31	53.91	54.42	31.22	37.52	30.09	37.02
Capana10g001412	25.91	15.11	11.76	12.42	24.46	28.19	31.36	24.58	29.64	30.59	21.27	21.86	16.74	17.88
Capana03g002053	65.87	49.26	46.94	39.26	40.09	107.39	64.72	62.98	10.16	33.31	5.08	9.02	11.19	18.86
Capana03g003013	21.41	11.36	7.88	8.27	7.66	14.22	16.39	14.2	8.97	7.95	5.88	5.39	6.05	23.12
Capana06g001351	0.39	0.16	0.16	0.00	0.09	0.13	0.12	0.38	0.16	0.04	0.07	0.04	0.00	0.11

Table S4. Primer sequences were used for qRT-PCR.

Gene name	Accession Number	Forward primer (5′→3′)	Reverse primer (5′→3′)
CaUbi3	AY486137	ATGGGTTCTGCTTCAATGG	TCATACTTTTTTACTGTTTGATGTTAG
CaFtsH06	Capana07g001803	ATGGCTACTTCATCAGTATGC	ATTAGGTGGATGAGTGTATGAAT
AtHSP101	At1G74310	TGCATTTAGCTGGTGCTTTGAT	CCACCGGCACTAGAGATTGC
AtAPX2	At3G09640	AATATGCTGCAGATGAGGATGC	CAAGAATCAAGGAGGTAGGAGATG
AtSOD1	At1G08830	TCAACTGGAAATATGCAAGCGAGGT	ACCACACAGCTGAGTTGAGCAAA
AtCAT1	At1G20630	AGCGCTTTCGGAGCCTCGTG	GGCCTCACGTTAAGACGAGTTGC
AtGPX3	AT2G43350	GGGTCAATCAGCGAGCTAC	CGATGGCGAAGAAGGGTATC
AtMYB44	AT5G67300	GGAACTGGTAACATCAGGAGGC	TTCTTTGCTCTCTTTCTGTATCCATC
AtRD29a	AT5G52310	TGTGCCGACGGGATTTG	CTGATGCCTCACCGTATCCA
AtDREB2A	AT5G05410	AAGGATTTGGGGTAAATGGGTTG	CAGCCTCATCATAAGCAGAAGCA
AtActin2	At3G18780	CTGTACGGTAACATTGTGCTCAG	CCGATCCAGACACTGTACTTCC

Table S5. Primers for subcellular localization of *CaFtsH06*.

Primer Name	Primer sequence (5'→3')	Enzymes
CaFtsH6	F: CGGGATCCATGGCTACTTCATCAGTATGC	*BamH I*
	R: GGGGTACCATTAGGTGGATGAGTGTATGAAT	*Kpn I*

Note: Highlighted text indicated the enzymes.

Table S6. The CDS sequence of *CaFtsH06* (Accession no. Capana07g001803).

```
ATGGCTACTTCATCAGTATGCATAGCAGGAAATGGTTTGTC-
TACACATAAAACACAGAAAGTCTTTAAGAAGGATGTTTATGGAAGGAAAATTTTATATTCCTCAAATCTTCCATCGTCTGGTAA
AACGTCAAGAGTAGTTGTAAAAGCATCTCTTCAGCAAAGGCCGGATGAAGGAAGAA-
GAGGCTTTCTTAAATTATTGCTTGGAAATGTTGGGCTTGGAGCGCCTGCTTTGTTAGGTAATGGAAAAGCCTATGCTGATGAGC
AAGGTGTTTCTAACTCAAGGATGTCTTATTCTAGATTTTTGGAATATCTGGACAAGGA-
TAGGGTGCAAAAAGTAGATTTGTTTGAAAACGGAACCATAGCTATTGTTGAGGCTGTATCTCCAGAATTAGGAAACCGGGTGCA
AAGAGTTAGGGTACAACTACCCGGGCTCAGCCAGGAACTTCTTCAAAAGTTCCGG-
GAAAAAAACATCGATTTTGCTGCTCACAATGCTCAAGAGGACTCAGGTTCTCTCATATTCAACTTGATTGGAAATCTGGCATTC
CCGCTTATTTTGATTGGTGGTCTTTTCCTGCTATCAAGGCGGTCTAACGGAGGAATGG-
GAGGTCCTGGTGGGCCTGGTAACCCACTAGCATTTGGTCAATCAAAAGCTAAGTTCCAAATGGAACCAAACACTGGTGTGACAT
TTGATGATGTTGCTGGTGTAGATGAAGCAAAACAAGATTTTATGGAGGTTGTAGAATTTTT-
GAAGAAACCTGAGAGGTTCACTGCAGTGGGGGCTCGTATTCCAAAAGGTGTTCTTCTTGTTGGTCCTCCTGGTACGGGGAAGAC
CCTGCTAGCAAAGGCAATTGCTGGTGAA-
GCGGGTGTTCCATTTTTCTCAATTTCAGGTTCAGAATTCGTCGAGATGTTTGTTGGTGTTGGAGCCTCTCGAGTCCGTGATCTT
TTCAAGAAGGCCAAGGAAAATGCTCCCTGCATTGTATTTGTTGATGAAATTGATGCTGTT-
GGGCGACAAAGAGGGACTGGAATCGGAGGAGGGAATGATGAAAGAGAACAGACCCTGAACCAACTATTGACGGAAATGGATGGT
TTTGAAGGAAATACTGGTATAATAGTTGTTGCAGCAACCAATCGTGCAGATATTCTCGAT-
TCGGCTTTGCTGTGACAACTGGTGCTGCTGGTGATTTGCAGCAGATCACCAGTTTGGCAAAACAGATGGTTGTCACTTTTGGGA
TGTCTGAACTTGGCCAGACCAGGACGCTTTGATAGACAAGTATCTGTGGATGTTCCAGA-
CATCAAGGGAAGAACAGAGATCTTAAAGGTTCATGCCGGCAACAAGAAGTTCGATTCAGATGTGTCTCTTGAAGTTATTGCCAT
GAGGACACCTGGCTTCAGTGGAGCAGATCTTGCTAACCTCTTGAATGAAGCAGCCATTCTT-
GCAGGTCGGCGTGGTAAAACAGCAATCGCATCCAAAGAGATTGATGATTCAATTGACAGGATAGTGGCTGGAATGGAAGGAACA
GTAATGACTGATGGCAAGAGCAAGAGTCTTGTGGCATATCACGAAGTT-
GGACATGCCATCTGTGGAACTCTCACTCCAGGGCATGATCCTGTTCAAAAGGTCACTCTAATCCCACGTGGTCAGGCAAAAGGT
TTGACCTGGTTCATTCCTGCAGATGATCCAACCTTAATATCCAAGCAGCAACTCTTCGCTA-
GAATTGTCGGTGGACTTGGTGGAAGAGCTGCAGAGGAAGTGATCTTTGGTGCACCTGAAGCCTGGTCACTCATGGATGCTTCAG
CCCAAAGTGGTGATGTAATCATGAGAATGATGGCCAGGAACTCAATGTCAGAAAA-
GCTAGCTGAAGACATCGATGCTGCTGTGAAGAGGCTTTCAGACAGTGCATATGAGATTGCGTTGAGCCAAATCCGCAGCAACCG
TGAAGCCATTGATAAGATTGTGGAAGTCCTCCTTGAAAAGGAGACGATGACAGGA-
GATGAATTCCGTGCTATTCTCTCAGAATTTGTGGAAATTCCTGCTAAACCCGTGTCCCTGCTGCTGTACCTACCCCAGCAGCCG
```

Table S7. Primers for gene silencing of *CaFtsH06*.

Primer Name	Primer sequence (5'→3')	Enzymes
CaFtsH6	F: CGGGATCCATTCCTCAAATCTTCCATCG	*BamH I*
	R: GGGGTACCTGCACCCGGTTTCCTAA	*Kpn I*

Note: Highlighted text indicated the enzymes.

Table S8. Primer pairs for transformation of *CaFtsH06*.

Primer Name	Primer sequence (5'→3')	Enzymes
CaFtsH6	F: CGGGATCCATGGCTACTTCATCAGTATGC	*BamH I*
	R: GGGGTACCATTAGGTGGATGAGTGTATGAAT	*Kpn I*

Note: Highlighted text indicated the enzymes.

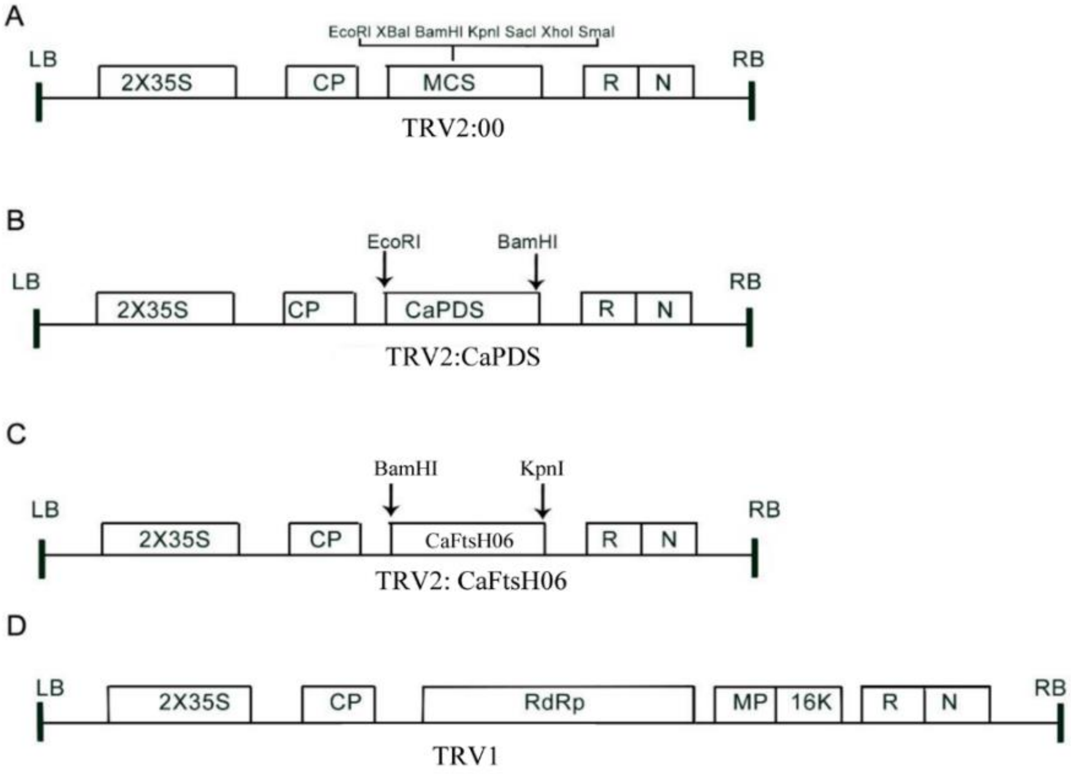

Figure S1. Structure of TRV2 vector for VIGS system. (**A**) the empty TRV2 vector; (**B**) TRV2:CaPDS vector; (**C**) TRV2:CaFtsH06 vector; (**D**) TRV1 vector. LB and RB: the left and right borders of TRV2 vector DNA; CP: coat protein; MP: movement proteins; 2 × 35S: two copies of the TRV2 with 35 promoter; RdRp: RNA-dependent RNA polymerase; 16 K: 16 KDa protein; MCS: multiple cloning sites; R and N: ribozyme and no-terminator.

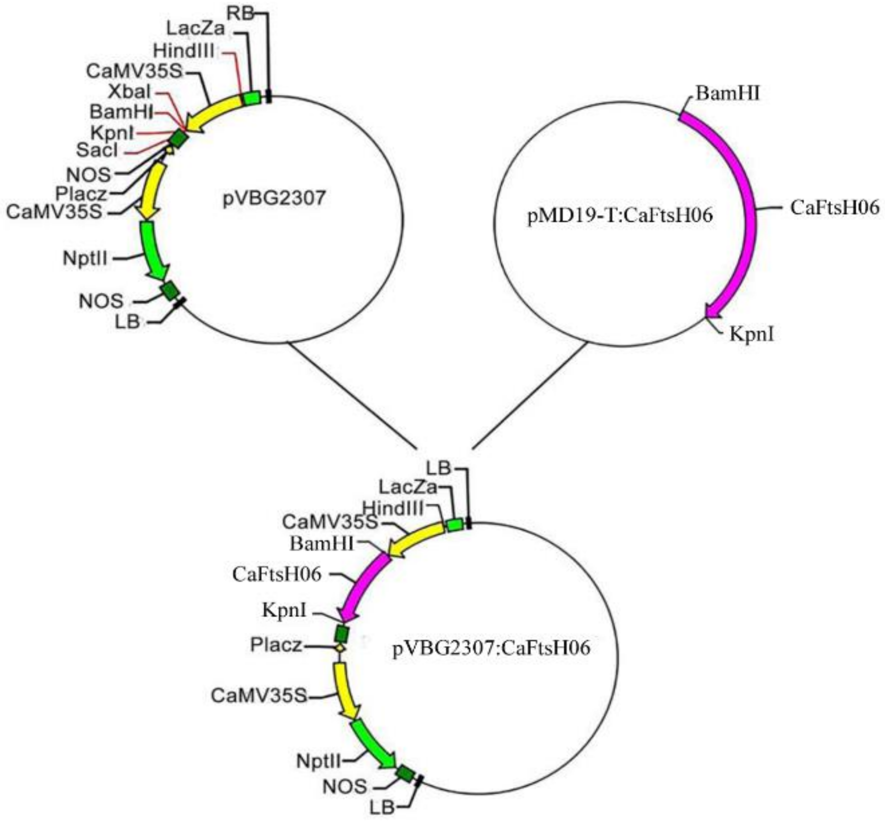

Figure S2. Structure of pVBG2307 vector for transgenic *Arabidopsis*.

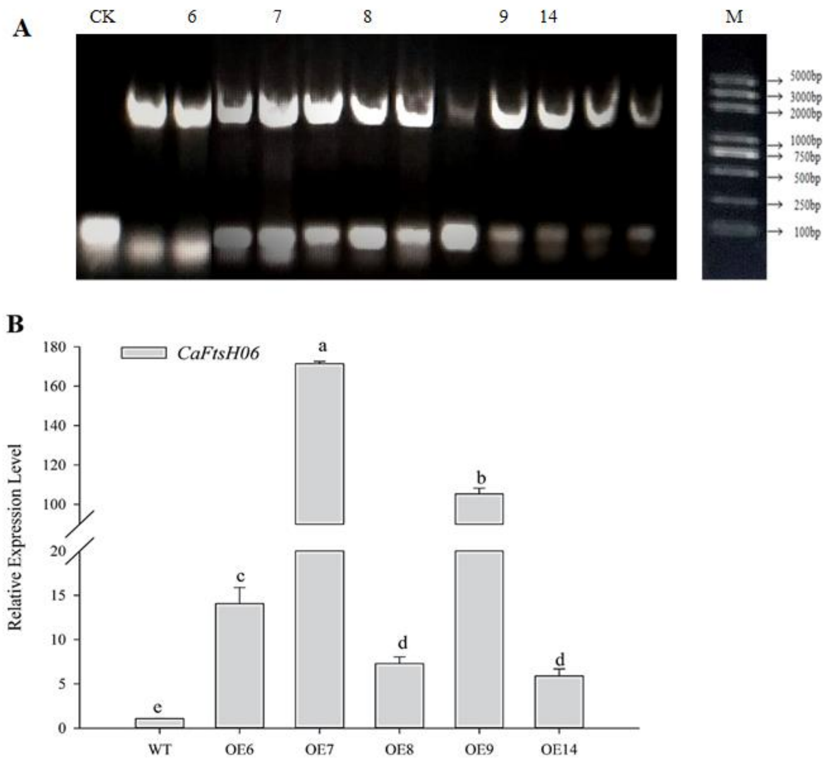

Figure S3. Detection and selection of the transgenic *Arabidopsis* lines. (**A**) PCR analysis of the *CaFtsH06* gene in wild type (WT) and *CaFtsH06*-OE *Arabidopsis*; (**B**) Relative expression level of WT and *CaFtsH06*-OE *Arabidopsis* lines (OE6, OE7, OE8, OE9, OE14) under normal conditions; The error bars represent SD for three biological replicates, and the lowercase showed the significant level at $\alpha = 0.05$.
